# Materials approaches for next-generation encapsulated cell therapies

**DOI:** 10.1557/s43579-024-00678-6

**Published:** 2024-12-02

**Authors:** Siddharth R. Krishnan, Robert Langer, Daniel G. Anderson

**Affiliations:** 1 David H. Koch Institute for Integrative Cancer Research, Massachusetts Institute of Technology, Cambridge, MA USA; 2 Department of Anesthesiology, Critical Care and Pain Medicine, Boston Children’s Hospital, Boston, MA USA; 3 Department of Chemical Engineering, Massachusetts Institute of Technology, Cambridge, MA USA; 4 Department of Biological Engineering, Massachusetts Institute of Technology, Cambridge, MA USA; 5 Institute for Medical Engineering and Science, Massachusetts Institute of Technology, Cambridge, MA USA; 6 Harvard-MIT Program in Health Sciences and Technology, Cambridge, MA USA

**Keywords:** Membrane, Biomaterial, Surface chemistry, Bioelectronics

## Abstract

Transplanted cells can act as living drug factories capable of secreting therapeutic proteins *in vivo*, with applications in the treatment of Type 1 diabetes (T1D), blood borne disease, vision disorders, and degenerative neural disease, potentially representing functional cures for chronic conditions. However, attack from the host immune system represents a major challenge, requiring chronic immunosuppression to enable long-lived cell transplantation *in vivo*. Encapsulating cells in engineered biomaterials capable of excluding components of the host immune system while allowing for the transport of therapeutic proteins, oxygen, nutrients, metabolites, and waste products represents a potential solution. However, the foreign-body response can lead to isolation from native vasculature and hypoxia leading to cell death. In this prospective article, we highlight materials-based solutions to three important challenges in the field: (i) improving biocompatibility and reducing fibrosis; (ii) enhancing transport of secreted protein drugs and key nutrients and oxygen *via* engineered, semipermeable membranes; and (iii) improving oxygenation. These efforts draw on several disciplines in materials’ research, including polymer science, surfaces, membranes, biomaterials’ microfabrication, and flexible electronics. If successful, these efforts could lead to new therapies for chronic disease and are a rich space for both fundamental materials’ discovery and applied translational science.

**Graphical Abstract:**

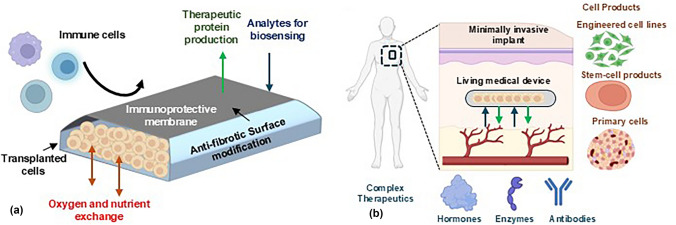

## Introduction

Small-molecule drugs have been the workhorse of the pharmaceutical industry but can only address a small fraction of currently known druggable targets. Recent advances have created new classes of drugs including peptides, proteins, and antibodies that have significantly expanded capabilities in biomedicine, but many of these novel drugs have unmet delivery challenges in stability and dosing.^[1]^ Here, the transplantation of live cells that can be used to produce proteins in a continuous or responsive manner offers a potential solution. As such, transplanted live cells can act as “living drug factories” *in vivo* that can address the twin challenges of stability and dosing. The transplantation of pancreatic islets for the treatment of Type 1 Diabetes (T1D) is an important example and has been the focus of significant research efforts for several decades.^[2,3]^ Successfully engrafted islet transplants can produce insulin in a glucose-responsive manner, and, in some cases, reduce or eliminate insulin dependence and hypoglycemic events entirely. The concept of cell transplantation can also work synergistically with advances in cell engineering to yield cell lines capable of producing many therapeutic proteins *in vivo*, with applications in the treatment of blood borne disorders such as hemophilia, degenerative neural disorders such as Parkinson’s disease,^[4]^ Alzheimer’s disease, and Huntington’s disease^[5]^ and in vision loss^[6]^ and the production of therapeutic antibodies. Taken together, the promise of cell therapies has generated significant excitement among patients and physicians for the treatment of T1D and a host of other chronic conditions requiring protein replacement.

However, despite this promise, host immune attack and transplant destruction represents a major challenge to cell transplantation, frustrating the widespread use of the therapy, and has been partially addressed by the use of chronic immunosuppressive therapies in the case of Type 1 Diabetes.^[7]^ Encapsulating cells in immunoprotective biomaterials represents a potential solution^[8,9]^ and broadly takes two forms: microencapsulation and macroencapsulation. The primary difference is in the number of cells transplanted; microencapsulation, often in spherical hydrogel or polymeric capsules, typically involves small numbers of cells or individual islets in each micro-scale capsule.^[10]^ Microcapsules offer favorable surface area to volume ratios, allowing for the diffusive transport of oxygen and nutrients. However, large numbers of capsules are required for therapeutic benefit, resulting in challenges in monitoring and retrievability. Macroencapsulation involves co-housing large, therapeutic doses of cells in single device constructs (‘macrodevices’), separated from host immune systems *via* semipermeable membranes capable of impeding the passage of host immune components while allowing the diffusive transport of key nutrients and metabolites. Macrodevices offer ease of monitoring and retrieval in case of adverse events, but in the absence of vasculature, can result in hypoxic conditions* in vivo*.^[11]^ These are further exacerbated by the formation of fibrotic tissue around implants. In addition to safety, retrievability and immune protection, two important factors will determine the translational potential of encapsulated cell therapies: first, the immune response and availability of nutrients can vary based on implant site. Subcutaneous sites are attractive for their ease of access and the potential for outpatient implantation and retrieval processes but exhibit strong immune responses to transplanted biomaterials^[12]^ leading to the formation of dense fibrotic tissue. In addition, they exhibit relatively low rates of fluid exchange and transport relative to intraperitoneal sites potentially lowering access to nutrients. Second, the transplantation of therapeutic doses of cells in small, patient-friendly sizes and form factors is an important challenge. For example, the treatment of T1D requires the transplantation of ~ 5,000 islet equivalents (IEQ)/Kg^[13]^ or 350,000 IEQ for a 70 kg adult, translating to a planar packing density of ~ 10,000 IEQ/cm^2^ or less for a size (~ 35 cm^2^ or less) comparable to existing implantable medical devices such as pacemakers or intrathecal pumps. In the absence of native vasculature, these packing densities can result in extreme hypoxia,^[14–16]^ leading to cellular death and transplant failure.

Progress towards long-lived, minimally invasive, immunosuppression-free cell encapsulation as a patient-friendly protein replacement therapy will depend critically on materials advances. In this prospective article, we will outline three broad areas for materials advances: (i) materials and surface modifications that can resist fibrosis; (ii) immune-protective, oxygen permeable membrane materials with tightly controlled, submicron pores; and (iii) materials to improve oxygenation and vascularization of transplants, all with a primary focus on macrodevices. Progress on these challenges will be supported by innovation in broad areas of hard and soft materials research, including in polymer synthesis, surface science, micro/nanofabrication, flexible electronics, and several others.

### Resisting fibrosis

The foreign-body response (FBR) is an inevitable consequence of any biomaterial or device material implant and has been implicated in a broad range of implant failures, from tissue engineered constructs to biosensors, drug delivery depots, and implanted cell therapy platforms. A complex cascade of immune events, culminating in the presence of a dense, relatively impermeable fibrotic capsule isolates the implant from the host vasculature**.** In subcutaneous sites, these processes can be similar to wound healing responses, resulting in the formation of significant scar tissue.^[12]^

**Figure 1:**
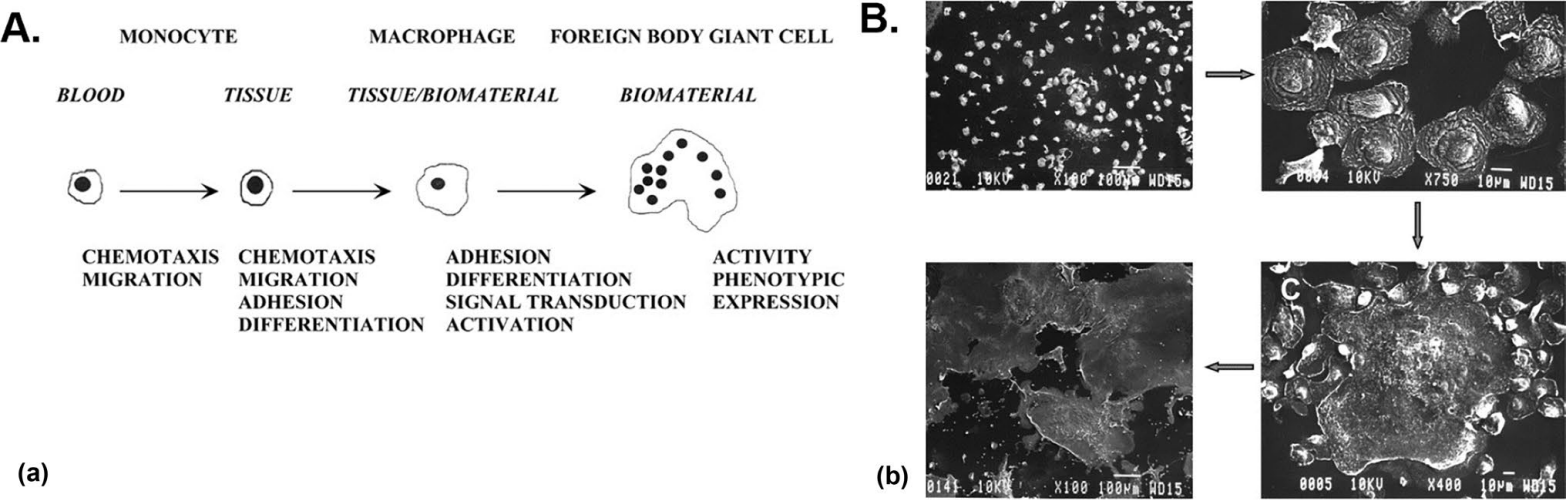
Immune response to transplanted devices. (a) Sequence of events involved in foreign-body response. (b) Optical images of progression of events on transplanted polyurethane surface leading to fibrotic tissue formation. Reproduced with permission from Ref. 66.

The precise mechanism of recognition is material specific, but often begins with nonspecific protein binding on the surface of the implant (e.g., fibrinogen, albumin),^[17]^ a process known as biofouling. These protein binding events serve as recognition mechanisms for macrophages. Macrophages are unable to directly attack and engulf large synthetic implants, and in response aggregate to form giant, nucleated foreign-body cells (FBCs) on the implant surface, leading to the production of chemokines, a class of signaling immune proteins. Fibroblasts are in turn recruited to the implant surface and induced to produce dense collagen to completely engulf the device surface in fibrotic tissue. As noted in Ref. 12, the low permeability of these dense fibrotic capsules possibly imparted an important evolutionary advantage in preventing systemic delivery of potentially poisonous or cytotoxic materials following wounds (Fig. 1). However, these same advantages are also responsible for significant barriers in the translation of encapsulated cell therapy devices, as they isolate devices from sources of oxygen and nutrients and impede the diffusive transport of secreted proteins. Materials-based approaches have attempted to target many of the above immune mechanisms to limit or altogether eliminate the FBR to realize “superbiocompatible,” immune-cloaked materials.

### Zwitterionic materials

**Figure 2:**
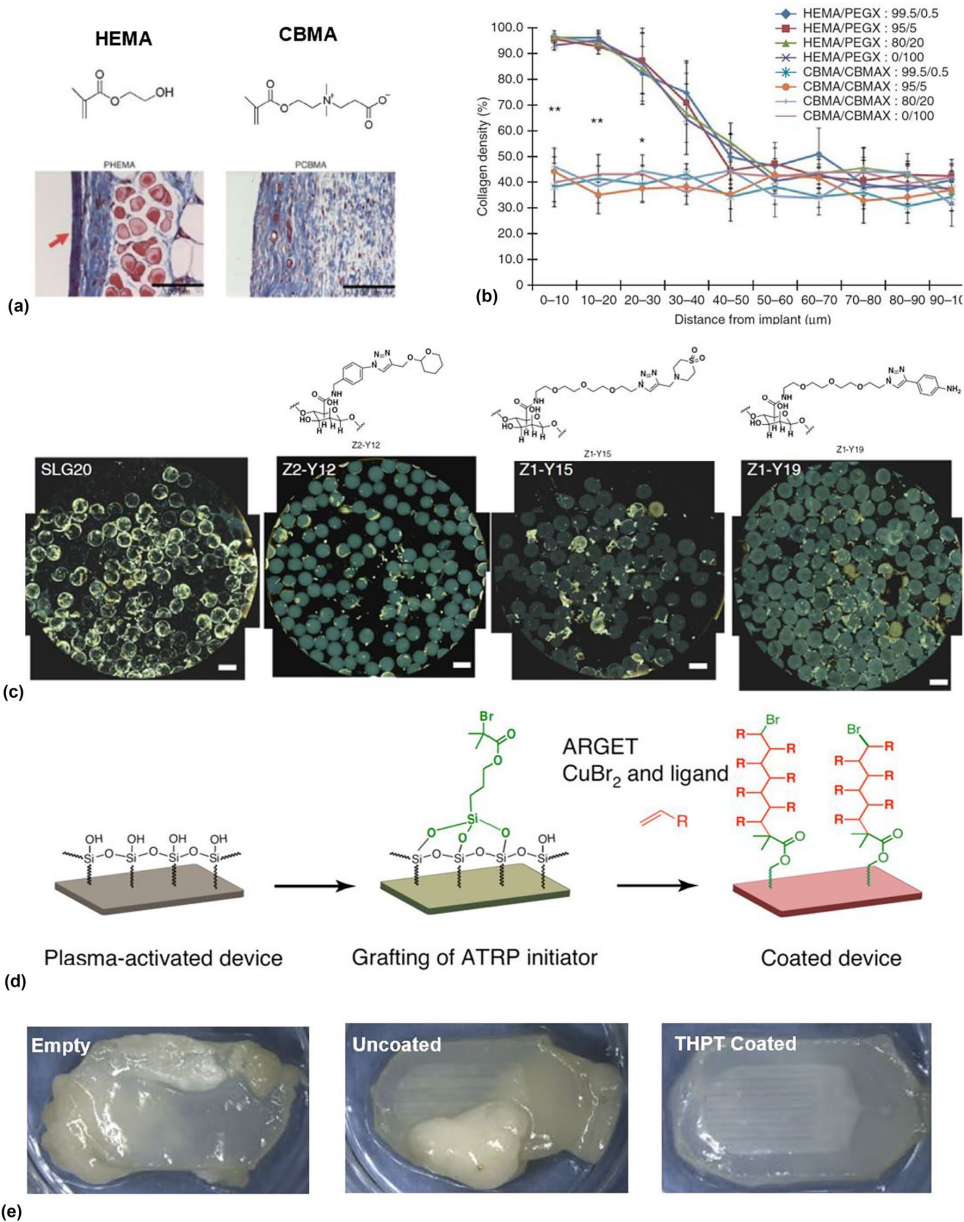
Anti-fibrotic surface modifications to enhance implant longevity. (a) Masson’s Trichrome stains of poly(2-hydroxyethyl methacrylate) (pHEMA) (left) and poly(carboxybetaine methacrylate PCBMA (right) 3 months after subcutaneous implantation (red arrow points collagenous deposition), with monomer structure over histological images. (b) Quantitative measurements of collagen density deposited on PHEMA an PCBMA as a function of distance from implant, for varying crosslinkers ratios. Reproduced with permission from Ref. 19. (c) Phase contrast imaging of retrieved devices from intraperitoneal sites in immunocompetent mice after 4 weeks, with SLG20 alginates modified with three lead triazole–thiomorpholine dioxide, (TMTD) materials, relative to unmodified alginate demonstrating reducing fibrosis. Reproduced with permission from Ref. 67. (d) Schematic illustration of surface modification procedures based on siATRP for macrodevices. (e) Bright-field images of retrieved devices from immunocompetent mice following 4-week implantation periods for empty (noncell-carrying), uncoated, and THPT-coated devices, respectively. Reproduced with permission from Ref. 27.

One important approach involves the use of surface modifications *via* zwitterionic polymeric materials, containing both positive and negatively charged groups in their chain. The resulting highly hydrophilic surface results in the formation of a strong, reconfigurable hydration layer to prevent nonspecific protein binding, a key early step in the immune cascade.^[18]^ An important material advance in this context is the development (poly) carboxy betadiene (PCBMA) in the form of a hydrogel.^[19]^ Implanting these materials in bulk hydrogel form in subcutaneous sites in immune-competent (C57BL/6 J) mice models for 3-month periods revealed two important effects, relative to PHEMA controls which are hydrophilic, and not zwitterionic. First, significant reductions in collagenous deposition [Fig. 2(a), (b)] around implant sites suggest reduced fibrosis. Second, the authors provide evidence for differences in macrophage polarization into their pro-inflammatory (in the PHEMA group) and their pro-angiogenic (PCBMA) states, respectively. The latter is particularly important, as it involves the formation of neovasculature around the implant site, suggesting a pathway to long-term cellular implant viability. Authors provide evidence of key markers associated with each group, including iNOS, IL-12, and TNF-α in the pro-inflammatory PHEMA group and MMR, Arg-1, and IL-10 in the pro-angiogenic PCBMA group. Phosphorylcholine is another important zwitterionic material,^[20,21]^ with derivatives such as methacryloyloxyethyl phosphorylcholine (MPC) demonstrating efficacy in reducing fibrosis in blood/serum contacting continuous glucose monitors to reduce measurement noise.^[22]^

### TMTD-modified alginates [Fig. 2(b)]

Another materials-based approach involves the modification of a commonly used biomaterial, alginate-derived hydrogels. Vegas, et. al., combinatorially screened over 700 alginate analogs based on modifications of alginate materials with alkynes, alcohols, and azides in subcutaneous sites C57BL/6 J mice and found 3 materials that successfully prevented fibrosis *in vivo*. All 3 materials had triazole linkages, based on Huisgen click chemistries, and 2 out of the 3 demonstrated enriched surface modifications relative to bulk hydrogels suggesting an important role for surface recognition processes. Critically, the team successfully expanded these concepts to nonhuman primates, demonstrating reduced fibrosis, a significant result in the field. In a related study,^[23]^ the team showed successful microencapsulation of xenogeneic stem-cell derived islets in streptozotocin (STZ)-induced diabetic C57BL/6 J mice with one of the lead materials (triazole–thiomorpholine dioxide, TMTD), resulting in diabetic reversal for > 170 days, and elevated human C-Peptide levels over the same period. Further expansion of these concepts led to the first demonstration of immunosuppression-free transplantation of pancreatic islets in nonhuman primates^[24]^ *via* immunoprotective TMTD-modified alginate hydrogels for 4 months. While an exact mechanism is not yet fully understood, these types of high-throughput screens *in vivo*, analogous to traditionally drug-discovery approaches, have considerable promise as an approach to finding anti-fibrotic materials. In this context, studies elucidating structure–function relationships for these kinds of materials will be vital in identifying future anti-fibrotic properties for future materials development and supporting additional large-throughput screens. Additional materials’ approaches highlight the potential for optimizing the size,^[25]^ shape, and surface topography^[26]^ of implants, but the latter effect has not been explored in the context of cell encapsulation technologies.

### Direct modification of macrodevice surfaces *via* small molecule grafting [Fig. 2(c)]

Each of the above approaches has been applied primarily in the context of microencapsulation. Bose, et al.,^[27]^ expanded these concepts to yield an anti-fibrotic THPT surface modification of polymeric device surfaces for fully retrievable, immunoprotective macroencapsulation devices (macrodevices). Here, macrodevices comprised immunoprotective polycarbonate (PCTE) membranes bonded to silicone bodies with channels formed *via* soft lithography. Silanization of all surfaces (both PDMS and PCT) followed by surface-initiated atom transfer radical polymerization (siATRP) yielded ~ 9% surface coverage of triazole-based polymer brushes. CuBr_2_ was used as a catalyst and removed with repeated washing cycles from solid surfaces with confirmation of surface chemistry from X-ray photoelectron spectroscopy. The reducing agent in the siATRP process was Sn (ii) 2-ethylhexanoate. Direct comparisons with well-known anti-fibrotic zwitterionic materials such as CBMA, PC [represented as R in Fig. 2(d)] and to unmodified devices surfaces demonstrated strong performance in inhibiting fibrosis in intraperitoneal implants in C57BL/6 J mice. Here, two key results highlight the promise of the technology: first, the *in vivo* ~ 130-day survival of encapsulated human embryonic kidney (HEK-293 T) cells edited *via* lentiviral approaches to produce erythropoietin (EPO), suggests pathways to protein replacement therapies based entirely on off-the-shelf cell lines. Dual findings of reduced fibrosis and enhanced serum protein levels in mouse groups containing TMTD-modified devices over the 130-day period are especially notable. Second, the demonstration of diabetic reversal in STZ-induced diabetic mice *via* the transplantation of pancreatic rat islets in the devices for 75 days suggest promise as a treatment for T1D. Notably, both the demonstrations involve xenogeneic transplantation without the use of immunosuppression, in fully immune-competent animal models.

Overall, the ability to modify surfaces to resist fibrosis across a range of transplant models, anatomical sites, and cell types remains an important unmet need. There exists significant promise in the field of superbiocompatible materials development, with important applications in encapsulated cell therapies, and also sensors, pacemakers, drug delivery devices, and neural probes.

## Immune-protective membrane materials

Cell-encapsulation devices that are designed to operate without immunosuppression require immunoprotective materials to protect transplanted cells from host immune systems. In traditional microcapsule technology, these demands have been achieved *via* crosslinked alginate hydrogels that are permeable to oxygen, nutrients, and metabolites, while preventing direct attack *via* immune components.^[25]^ These microcapsules are often transplanted in spherical form factors, which provide additional benefits in surface area to volume ratio, allowing for improved diffusion. However, the need for easy monitoring and retrievability has also motivated the development of materials for the immune protection of large collections of cells, potentially at therapeutic doses.^[10]^ The choice of immune-protective membrane materials is dictated by several key factors: (i) pore size and density; (ii) permeability to oxygen; (iii) permeability to key metabolites and nutrients; (iii) size dispersity and organization of pores; (iv) mechanical strength and robustness; and (v) biocompatibility. Several classes of inorganic and polymeric materials have been explored in this context, each with a distinct set of advantages and disadvantages.^[28]^ Broadly, inorganic materials such as silicon^[29]^ utilize advantages in thin-film planar processing, with pores that can be small and tightly controlled, and with thin membranes. However, they can also be brittle, costly, and often support only a limited number of pore geometries such as squares and rectangles and at low densities. In addition, they tend to be relatively oxygen impermeable, with oxygen transport occurring only through pores. Other inorganic materials such as titania and alumina remain to be studied extensively in *in vivo* settings to assess their biocompatibility, mechanical performance, and immunisolation properties. In contrast, polymeric membranes offer advantages in ease of processing, cost, throughput, pore density, and oxygen permeability but also represent challenges in controlling pore sizes. In addition, the increased thickness of polymeric membranes (> 10 µm) can provide additional resistance to diffusive transport. Despite ongoing challenges, polymeric membranes have been extensively used in cell encapsulation devices, including in multiple human studies.

### Inorganic materials [Fig. 3(a)]

**Figure 3:**
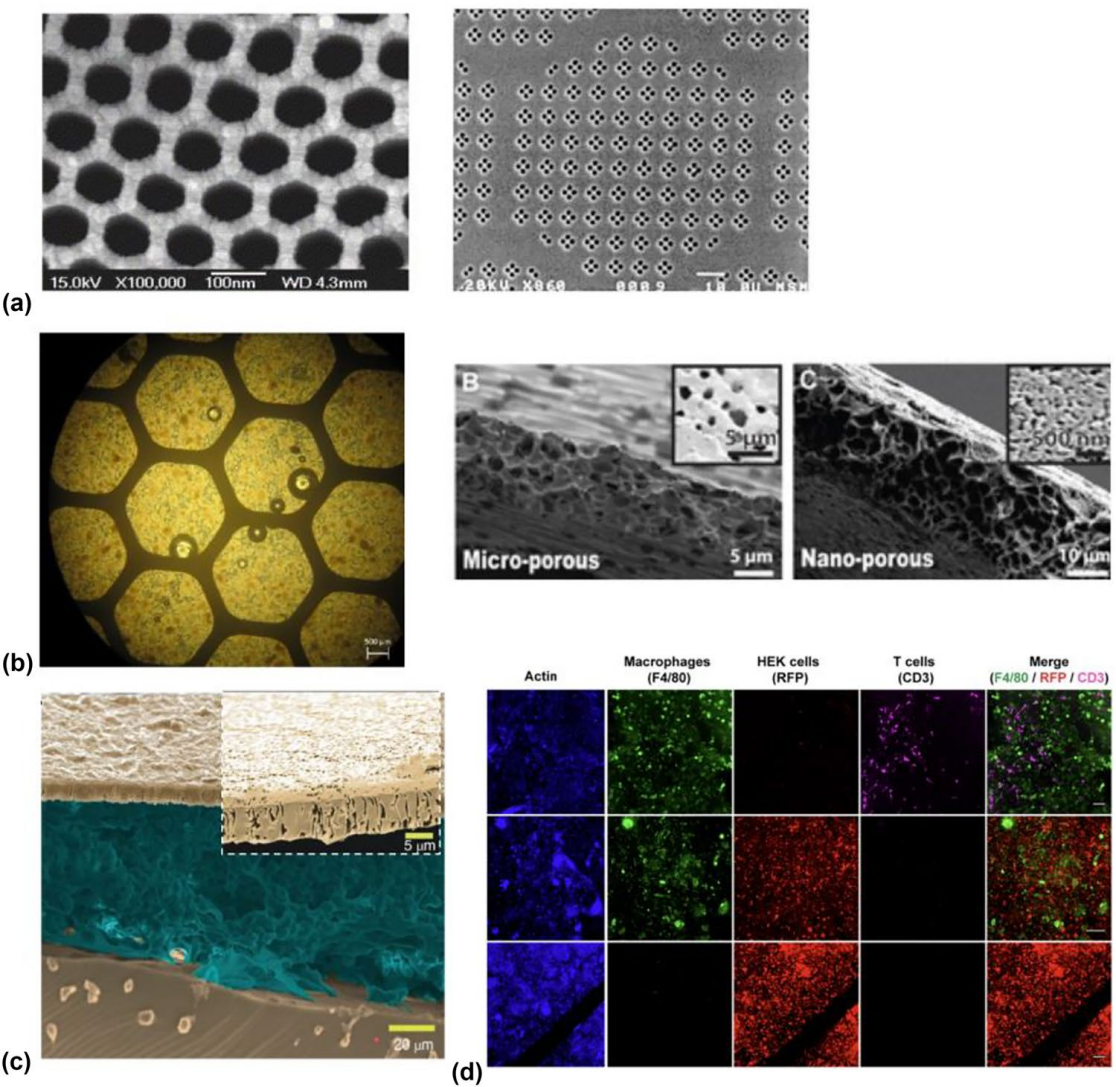
Materials approaches to immune-protective membrane development: (a) Nanoporous inorganic materials include anodized alumina (left, reproduced with permission from) Ref. 35 and silicon (right, reproduced with permission from) Ref. 68. (b) Polymeric materials include polytetrafluorethylene (left, with alginate filling) Ref. 69 and microporous and nanoporous polycaprolactone (right, Reprinted with permission from Ref. 33 Copyright 2015, American Chemical Society. (c) Polycarbonate membranes with micron-scale and submicron pores (inset). (d) Fluorescent images demonstrating nonintuitive immunoprotective effects of polycarbonate pores *in vivo* at 1 µm diameters, impeding the transport of T-Cells but allowing passage of macrophages without affecting transplanted cellular function (HEK293T cells). Reproduced with permission from Ref. 27.

Expanding approaches in microfabrication and micro/nanoelectromechanical systems (MEMS/NEMS) to produce submicron immunisolation features has been heavily explored and featured standard semiconductor materials such as silicon and dielectrics. Silicon-based membranes have received considerable attention. A well-studied process is based on the selective etching of a sacrificial thermally grown silicon dioxide layer, resulting in pore sizes down to 18 nm, with tolerances of 0.5 nm and distributions of 5%^[30]^, with some demonstrated advantages in immune-isolation.^[31]^ Other investigated inorganic materials include alumina and titania.^[32]^

### Polymeric materials [Fig. 3(b), (c), (d)]

Several porous polymeric materials have been explored for cell macroencapsulation. Polytetrafluorethylene (PTFE) has been used in preclinical and clinical studies, with larger pores (> 1 µm) supporting vascularization and smaller pores (0.4 µm) for immune protection. These membrane materials cover cell-housing chambers that can be filled with cellular components, typically in hydrogel matrices. Polycaprolactone (PCL) has been deployed for its ease of fabrication, biocompatibility, and biodegradability, with potential device lifetimes that can be designed to coincide with desired transplant lifetimes.^[33]^ Both microporous (~ 2 µm pores) and nanoporous (< 100 nm) membranes were evaluated with fluc expressing MIN6 cells and demonstrated glucose responsiveness in *in vitro* models, and viability over 90 days in balb/c mouse models.

An important recent effort demonstrated nonintuitive size effects^[27]^ in track-etched polycarbonate (PCTE) membranes in xenogeneic transplant models, and elucidated upper limits on possible pore diameters in immune-protective membranes. Here, PCTE membranes were bonded to soft-lithographically fabricated PDMS device bodies *via* silanization and UV-treatment, and transplanted in intraperitoneal sites and loaded with HEK293T cells transformed to produce erythropoietin (EPO). Interestingly, pore diameters as high as 1 µm continued to support transplanted cell function, as measured by serum EPO concentrations. Interestingly, at 3 µm pore diameters, both T-cells and macrophages were observed inside the device body following explanation at 5 weeks, but at 1 µm, only macrophages were observed, and HEK-cell function was preserved [Fig. 3(c), (d)]. Pore sizes of 0.8 µm and smaller resulted in no immune cell infiltration. PCTE-membrane devices have also been shown to be compatible with oxygen-generating bioelectronic devices.^[34]^

Taken together, while there has been important progress in the design and assessment of immune-protective, semipermeable membrane materials, significant additional challenges remain in ensuring a highly oxygen permeable, tightly controlled, monodisperse, porous niche for cell encapsulation, with poor pore size control over large areas across a broad spectrum of materials (polycarbonates, silicones) representing an important unmet need. Other important considerations such as materials’ compatibility with existing device materials, the ability to modify surfaces to resist fibrosis and mechanical performance over long-term implantation will play a critical role in determining a clinically viable membrane technology. This remains an important area of materials’ research that has the potential to significantly advance cell encapsulation therapies.

## Materials for improving oxygenation

As noted above, oxygenation represents a key limitation of artificial cell encapsulation devices, owing to a lack of contact of transplanted cells with native vasculature. While the concentration of dissolved oxygen in arterial blood is 11–12%, the corresponding value for a densely packed islet cluster can be 1% or lower,^[14]^ resulting in severe hypoxia, leading to loss of cell function and cell death. These challenges are further exacerbated in the presence of fully formed fibrotic capsules that isolate transplanted cells from host vasculature. While several approaches to improving oxygenation have been demonstrated, they can broadly be divided into two categories: (i) direct delivery of oxygen to transplants and (ii) promoting vascularization around the graft. Both approaches have relied critically on materials’ advances.

### Direct oxygen delivery

**Figure 4:**
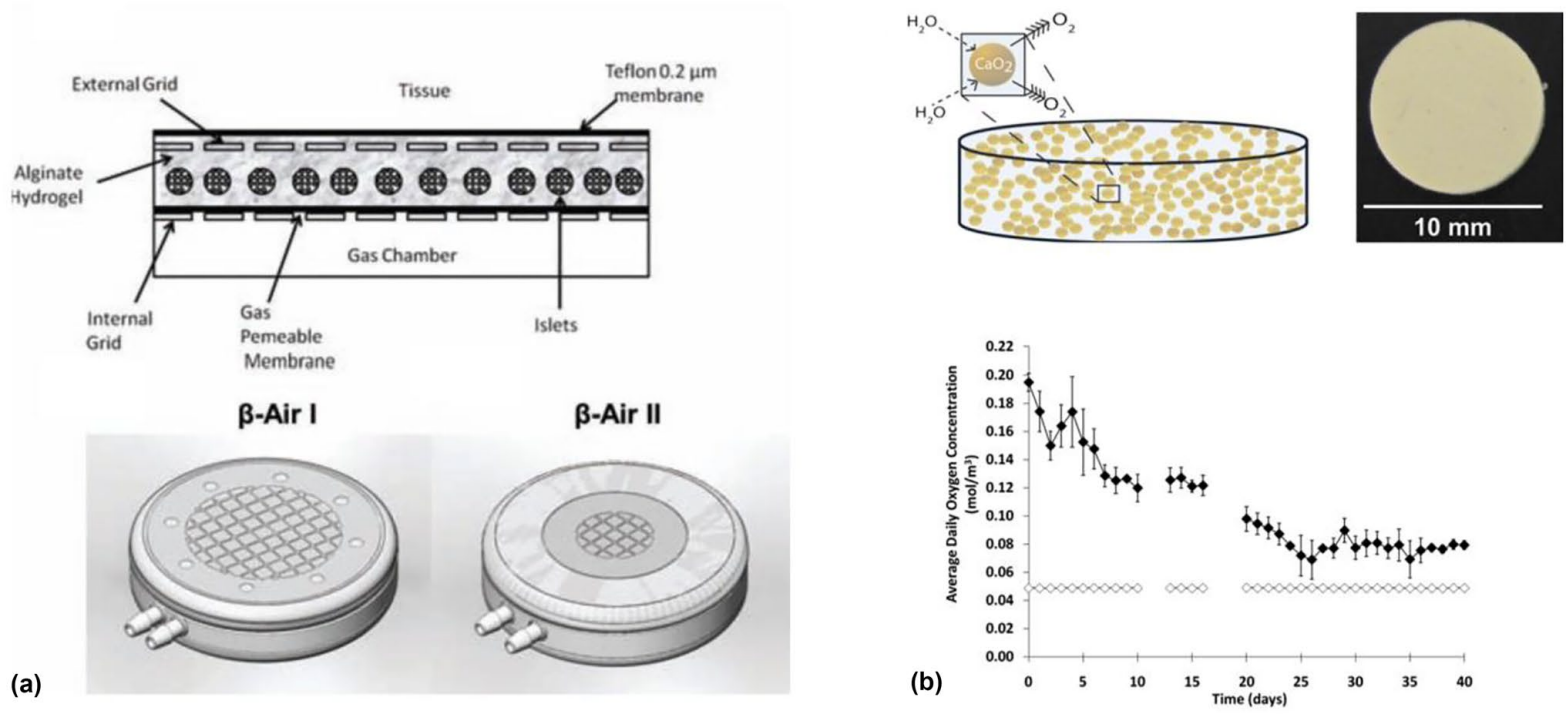
Direct oxygenation strategies. (a) β-Air Immune-protective macrodevices from β-O_2_ technologies with transcutaneous ports for direct oxygen delivery and PTFE immunoprotective membranes. ^[69]^ (b) Calcium oxide-PDMS composite material for hydrolytically activated oxygen generation (top), with oxygen profile over 40 days*in vitro*.^[39]^

Beta O_2_, a company commercializing the “β-Air” platform for pancreatic islet transplantation without immunosuppression, provided an important early advance in direct oxygenation of cellular transplants. The technology relies on subcutaneously implanted cell-housing chambers containing pancreatic islets in an alginate tissue-slab configuration [Fig. 4(a)], separated from the host immune system *via* immune-protective multilayer polytetrafluorethylene (PTFE) membranes. The core of the technology involves the daily delivery of oxygen *via* transcutaneous, refillable ports to address the well-known phenomenon of islet hypoxia in subcutaneous sites. The platform demonstrated several key proof-of-concept results that suggest the feasibility of immunosuppression-free cell transplantation, including complete diabetic reversal for 90-day periods *via* subcutaneous sites and high-density (4,800 IEQ/cm^[35]^) loading configurations. In further studies, the group demonstrated long-term xenograft survival in nonhuman primates, where pig islets were subcutaneously transplanted in β-air devices for 9 months without immunosuppression.^[36]^ Significantly, the group also showed allogeneic islet survival in first-in-human studies following extended implantation, in spite of not at a dose sufficient to achieve insulin independence in transplanted patients.^[37]^

The use of oxygen-generating biomaterials to directly oxygenate cells *in vivo* represents another approach.^[38]^ An important study demonstrating the feasibility of direct oxygenation approaches involves the use of hydrolytically activated, oxygen-generating biomaterials is detailed in Ref. 39 Here, calcium oxide (CaO_2_) is used as a hydrolytically active oxygen-generating material: when brought into contact with liquid water (as found in the body), it forms oxygen based on a reaction given by 2CaO_2_ + H_2_O→2Ca(OH)_2_ + 2H_2_O→2Ca(OH)_2_ + 2H_2_O + O_2_. Incorporating CaO_2_ into a common, medical grade, permeable silicone such as polydimethylsiloxane (PDMS) creates bulk materials that create oxygen on contact with water [Fig. 4(b)]. *In vitro* studies with β cell lines (MIN6) and primary pancreatic islets in hypoxic conditions demonstrate the efficacy of these materials in maintaining cell viability (as measured by decreased lactose dehydrogenase production relative to controls) and metabolic activity (as measured by MTT assays). An ongoing challenge with oxygen-generating biomaterials is enhancing the longevity of these implants following the depletion of the reactant mass.

### Electrochemical oxygen generation materials

**Figure 5:**
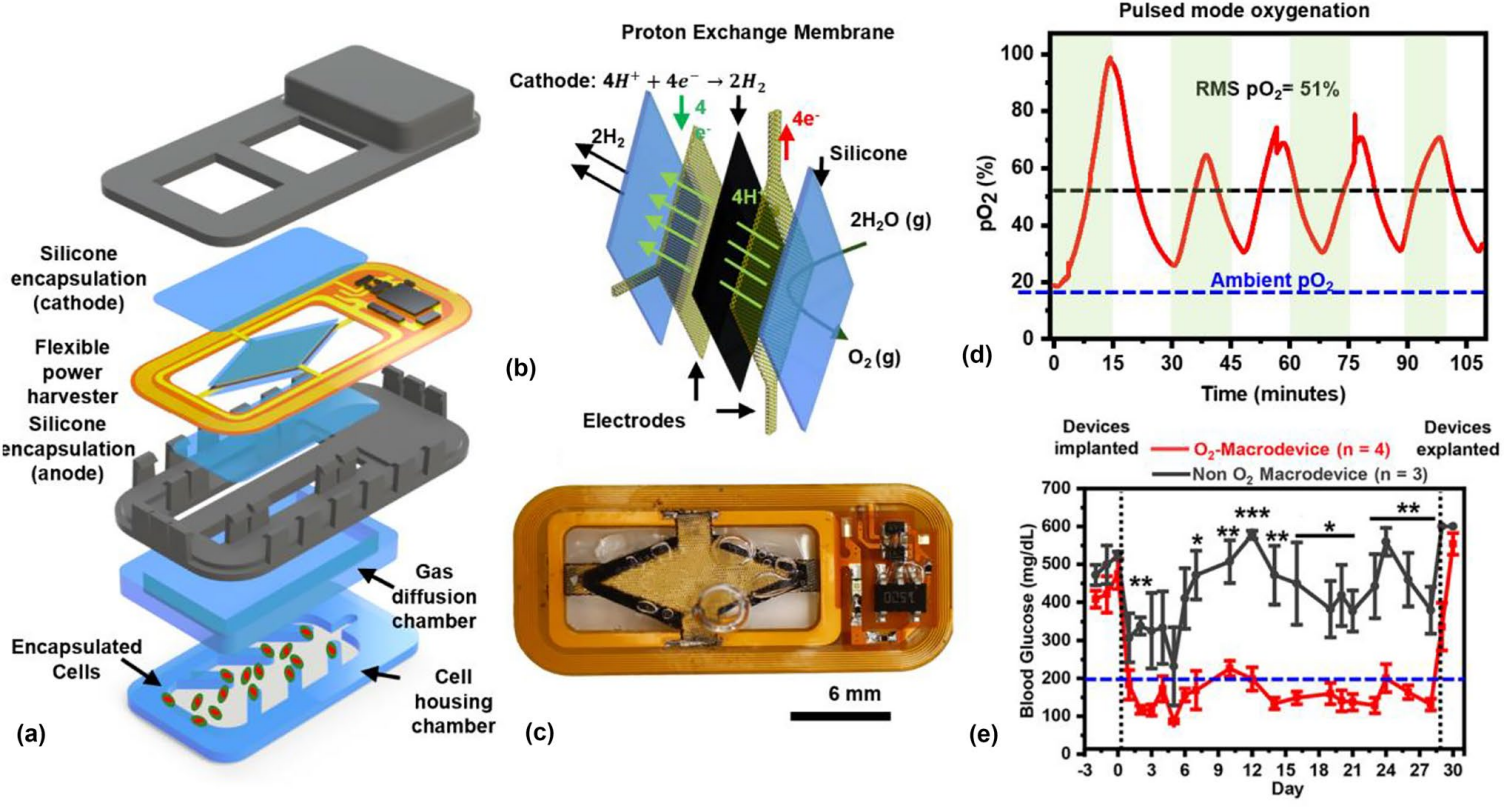
Wireless, battery-free electrochemical oxygen generation. (a) Multilayer exploded view schematic demonstrating device construction. (b) Schematic of proton exchange membrane (PEM) electrolysis based on poly[perfluorosulfonic acid] and silicone encapsulation layers. (c) Optical image of wireless battery-free device generating oxygen and hydrogen bubbles. (d) Oxygen modulation inside device *via* pulsed-mode operation. (e) Diabetic reversal in diabetic immune-competent mice transplanted with xenogeneic (rat) islets in subcutaneous sites over 1 month with electrochemical oxygen-generating devices. Reproduced from Ref. 34.

Recent work from our group^[34]^ seeks to address the oxygenation challenge *via* direct electrolytic splitting of water in the body in a fully wireless, battery-free bioelectronic device, a concept first proposed in Ref.^[40]^. Our system relies on several key materials’ subassemblies (Fig. 5). At its core, our device relies on a perfluorinated ionomer-based proton exchange membrane (PEM), commonly used in the fuel-cell industry to produce hydrogen from seawater *via* PEM electrolysis. When a voltage greater than the water-splitting voltage (1.2 V) is applied to the surface of the PEM, water is split at the anode, with protons (H^+^) driven across the PEM due to its high selectivity to proton transport, and it recombines with electrons at the cathode to form molecular hydrogen, which is allowed to dissipate into the body. Here, the production rate (~ < 25 nmol/s) of H_2_, and potential anti-inflammatory properties^[41,42]^ support safety considerations for long-term transplantation. Standard catalytic materials such as Iridium-Ruthenium Oxide (anode) and Platinum-Black (cathode) integrated onto the PEM *via* standard deposition methods (spray coating, dip casting) support high electrolytic currents. The PEM system is encapsulated in PDMS, owing to its permeability to water, oxygen^[43]^ and hydrogen,^[44]^ and to suppress parasitic reactions such as Cl_2_ formation at the anode by frustrating transport. An important advance in this context is the use of water vapor, rather than liquid water as the reactant feedstock, as first shown in Ref. 45, to obviate the need for liquid handling systems *via* entirely diffusive transport. Hydrogen formed as a cathode byproduct at relatively low rates (< 10 nmol/s) was allowed to dissipate into the body. Entirely battery-free power transfer is another important advance, obviating the need for recharging and providing important advantages in size. Resonant inductive power transfer has been used in several recent demonstrations of fully implantable devices for optogenetic neural stimulation,^[46]^ drug delivery,^[47]^ and recording.^[48]^ Here, an inductive coil structured directly into a flexible circuit board forms the basis of an LC-oscillator circuit designed to oscillate at 13.56 MHz. When combined with electronic components for voltage rectification and regulation, the resulting LC circuit can power an electrochemical reaction based on an externally located primary transmitter coil. Combining PEM and power harvesting assemblies with soft-lithographically defined silicone layers for gas transport and cell encapsulation, along with immune-isolating membranes, yields a fully integrated device suitable for *in vivo* studies. Electronic subassemblies and PEM electrolyzer systems were cast in PDMS to prevent direct contact with biofluid. Demonstrations in subcutaneous sites, with devices implanted *via* minimally invasive surgeries, in immune competent, freely moving animals suggest key capabilities. Studies with encapsulated HEK-EPO cell lines demonstrated elevated protein production over 1-month studies relative to non-oxygenated controls. Importantly, studies involving pancreatic rat islet transplantation in STZ-diabetic C57BL/6 J mice demonstrated insulin independence and diabetic reversal over 1-month period, with glucose-responsive islets at high loading densities (~ 1000 IEQ/cm^2^). *In vitro* efforts suggest pathways to increase loading densities to ~ 5000 IEQ/cm^2^ or greater. Non-proton exchange membrane based electrolytic approaches relying on catalyst design to enhance anodic oxygen evolution reactions also show promise in mitigating hypoxia and enabling cell viability *in vivo*, when exposed directly to biofluid.^[49]^

Other promising materials’ approaches to direct oxygen delivery involve the use of porous, oxygenated scaffolds^[50]^ and lithium-based materials’ system for the capture of carbon and reconversion into oxygen, as a mirror image of respiratory processes.^[51]^

### Vascularizing materials

The absence of native vasculature represents a critical cause of transplanted cells’ failure, resulting in limitations in oxygen and nutrients. Accordingly, significant research efforts have focused on decreasing diffusion distances between cells and neovasculature in transplant sites, ideally to < 300 µm. Innovative early work in the area was *via* Baxter, through its Theracyte platform,^[52]^ based on bi-layer polytetrafluoroethylene (PTFE) membranes. Here, the use of relatively large pores (> 1 µm) on the outer membrane layer allowed for complete infiltration by immune cells, followed by a process of remodeling and revascularization. A second, immunisolating membrane (pore size < 0.02 µm) laminated to the first provided protection to cells. *In vivo* results in rats following 1-year subcutaneous transplantation revealed 80–100-fold increases in levels of vascularization in large-pore structures relative to controls. In addition, the infusion of vascular endothelial growth factor (VEGF) could provide a further increase in vascularization levels, leading to increases in insulin kinetics.^[53]^

More recent work on vascularizing materials is *via* Viacyte, through its Encaptra platform that is aimed at addressing a key unmet need in pancreatic islet replacement, namely finding a reliable supply of cells. Recent advances in stem-cell differentiation (recognized with the 2012 Nobel Prize in physiology or medicine for Shinzo Yamanaka and John Gurdon) can potentially allow for the generation of insulin-producing β-cells derived directly from stem-cell progenitors. These can be human embryonic stem-cell (hESCs)-induced pluripotent stem-cell (iPSC) lines. *In vivo* differentiation into fully mature, insulin-producing cells relies on oxygen tension^[54]^ and access to nutrients, suggesting the need for direct vascularization. In this context, encapsulating stem-cell progenitor cells in macrodevices allows for monitoring and retrievability in case of adverse events but is associated with oxygenation and nutrient access challenges. Recent clinical results^[55]^ demonstrate the platform’s capabilities in facilitating immune protection (pore size ~ 0.45 µm), with good safety profiles, minimal adverse events and evidence of insulin, glucagon, and NKX6-1 (a transcription factor in β-cells) from hESC lines (pancreatic endoderm cells, or PEC) *in vivo*,^[56]^ but subsequent fibrosis and hypoxia remain an impediment to long-term viability. A follow-on study, in collaboration with Gore, a well-known materials’ company to improve membrane performance, demonstrated promising results but failed to achieve insulin independence. More recent efforts involve a non-immunoprotective, pro-vascularizing device that allows direct contact between neovasculature and encapsulated cells, *via* perforated, “open” layouts known as “PEC-Direct.” Here, the *in vivo* and clinical studies involve the use of immune suppression, but retain advantages in retrievability and ease of monitoring, and are aimed primarily at subcutaneous implantation sites for straightforward surgeries.

Clinical results with PEC-Direct have been notable. A pair of 2021 studies^[57, 57]^ first described subcutaneous implantation of these devices as part of a phase 1/2 open-label study, involving 15 and 17 subjects, respectively. Key results here include device safety, insulin production, and glucose-responsive C-peptide secretion of encapsulated PEC cells derived from Cyt49 cell lines, in PEC-direct, aided by vascularization, though without reductions in exogenous requirements and at subtherapeutic doses. These results suggest the feasibility of PEC cells dividing into functional insulin-producing β-cells in humans when encapsulated in macrodevices.

**Figure 6:**
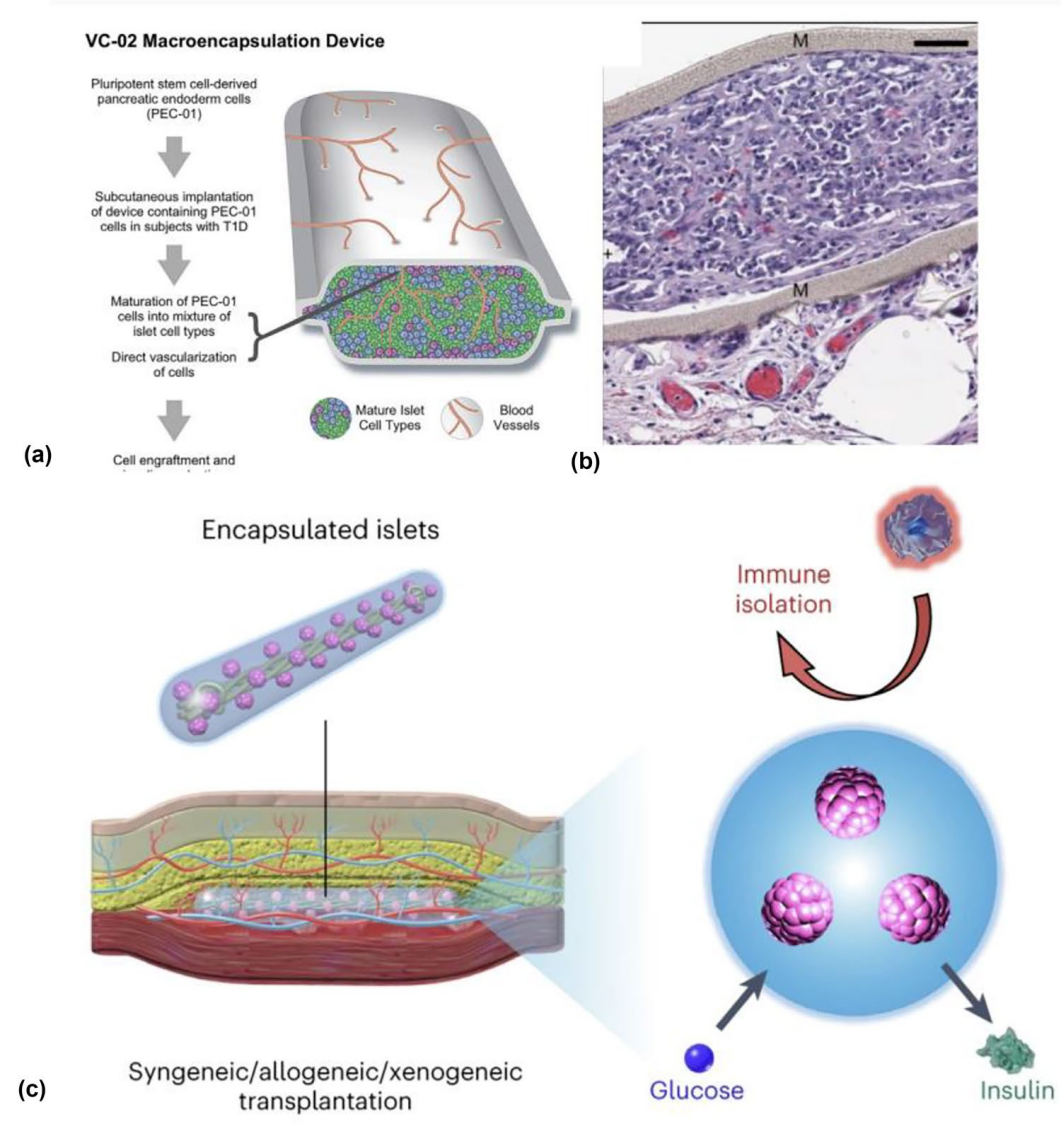
Vascularizing materials for cell transplantation. (a) PTFE-based nonimmunisolating “PEC-Direct” devices for the transplanting pancreatic endoderm cells from Viacyte Inc. Reproduced with permission from Ref. 57 (b) Histological stains on retrieved devices highlighting the formation of vasculature (red erythrocytes) Reproduced with permission from Ref. 57 (c) Nylon-hydrogel macrodevices transplanted into subcutaneous sites pre-vascularized *via* the prior implantation of nylon catheters for immune protection and nutrient. Transport. Reproduced with permission from Ref. 64.

A more recent study describes important clinical advances in the PEC-direct platform based on interim results from the same study, on a subset of patients with higher (2–threefold) doses of cells,^[59]^ based pro-vascularizing devices based on similar open layouts to those described above (Fig. 6).^[60]^ Here, 4 out 10 patients exhibited detectable C-Peptide levels 6 months following transplantation, with 3 of these patients achieving > 0.1 nmol/l. Significantly, one patient, who also had the highest C-Peptide concentration exhibited improved glycemic control, with time spent in range (as measured *via* CGM) improving from 55 to 85%. Histological images of the devices, demonstrating erythrocytes, are in Fig. 6(b). These results suggest the promise of pro-vascularizing materials in improving glycemic outcomes in patients and providing a potential translational pathway to stem-cell derived products in subcutaneous sites. However, despite these results, two important challenges remain: first, the β-cell mass in these devices was < 5%, significantly less than that of α-cells (16%), suggesting the need for additional materials’ advances to improve differentiation *in vivo*; second, patients required immune suppression throughout the course of the study, with immune protection of cells remaining an important unmet need.

Several studies have exploited “pre-vascularized” sites for subcutaneous implantation of pancreatic islets.^[61–63]^ Here, subcutaneous sites are prepared by the implantation of biomaterials that are known to promote wound healing and vascularization for several weeks. Following biomaterial retrieval, islets are then infused into the space left behind, exploiting the highly vascularized niche. These approaches have suggested long-term viability and access to oxygenation and nutrients but require immunosuppression. An innovative approach was recently proposed in Ref. 64, combining immune-protective macrodevices and prevascularization in multiple contexts, including syngeneic (balb/c islets transplanted into balb/c recipients), allogeneic (balb/c islets transplanted in C57BL6/J mice), concordant xenogeneic (rat islets in C57BL/6 J mice), and discordant xenogeneic (human islets in C57BL/6 J mice) models, with strong results. The subcutaneous site was prepared *via* the insertion of a nylon catheter for 4–6 created the required niche, followed by explantation to leave behind a highly vascularized, cylindrical site. The devices, named “subcutaneous host-enabled alginate thread” or SHEATH, are an expansion of concepts first proposed in Ref. 65 comprised nylon threads twisted into each other into helical structures, and embedded in poly(methyl methacrylate)/N, N-dimethylformamide in solutions containing CaCl_2_, for stable interfaces into which to load islets. Further embedding in crosslinkable alginate hydrogel molds (with crosslinking *via* Ca^2+^ ions) provided an immunoprotective, cylindrical outer hydrogel surface. The resulting device was loaded directly into the shape-matched pre-vascularized subcutaneous niche [Fig. 6(c)]. *In vivo* testing revealed functional diabetic reversal in allogeneic models (~ 180 days) and concordant (~ 190 days) xenogeneic models. The transplantation of human islets resulted in partial diabetic reversal (blood glucose levels ~ 300 mg/dl after 30 days), suggesting some engraftment. Notably, the authors demonstrate that these types of platforms can be easily retrieved and swapped for functional grafts in case of transplant failure owing to easily accessible subcutaneous transplantation site.

The continued development of pro-vascularizing materials with immune-protective membranes in conjunction with the delivery of angiogenic factors holds considerable promise for the continued development of encapsulated cell therapies.

## Conclusion

Encapsulated cell platforms are a form of biohybrid living medical devices that have the potential to revolutionize protein replacement therapies with a broad range of applications. Materials advances can provide important breakthroughs in accelerating these therapies towards the clinic by promoting transplanted cell viability *in vivo* through reduced fibrosis, improved immune protection and oxygenation. Successful cell encapsulation can pave the way for off-the-shelf therapies where engineered allogeneic or xenogeneic cell lines engineered to secrete any protein of choice can be transplanted in patients in minimally invasive sites *via* outpatient procedures. In many cases such as in T1D, these technologies can lead to functional cures for chronic disease, significantly improving patient outcomes. We have discussed three areas with extensive possibilities for materials innovation: anti-fibrotic, superbiocompatible surfaces; nanostructured immunoprotective membranes; and the enhancement of oxygenation and nutrient transport. The future development of anti-fibrotic materials will rely critically on feedback between fundamental studies elucidating structure–function relationships, and high-throughput screens that identify best-performing materials. The use of machine learning tools to predict anti-fibrotic properties could also be an important area for future research, enabled by data from large *in vivo* screens. Next-generation immunoprotective materials’ development will rely on advances in micro/nanofabrication and additive manufacturing, allowing for the development of 3D, tissue-like constructs that can potentially obviate the need for immune suppression *via* tightly controlled nanopores. Finally, new oxygenation and vascularization strategies will be required to make careful tradeoffs between transplant size and power management, while also limiting the use of pro-fibrotic or pro-inflammatory materials. Advances in these three areas could drive significant growth in the field of encapsulated cell therapies.

## Data Availability

This review article is based on data from previously publishes studies and no new data were produced for this work. All referenced material can be found in cited sources at the end of this work.
